# Aerial drone observations identified a multilevel society in feral horses

**DOI:** 10.1038/s41598-020-79790-1

**Published:** 2021-01-08

**Authors:** Tamao Maeda, Sakiho Ochi, Monamie Ringhofer, Sebastian Sosa, Cédric Sueur, Satoshi Hirata, Shinya Yamamoto

**Affiliations:** 1 Wildlife Research Centre, Kyoto University, 2-24 Tanaka-Sekiden-cho, Sakyo, Kyoto Japan; 2 Institute for Advanced Study, Kyoto University, Yoshida Ushinomiya-cho, Sakyo, Kyoto Japan; 3 Université de Strasbourg, CNRS, IPHC, UMR 7178, Strasbourg, France; 4 Institut Universitaire de France, Paris, France

**Keywords:** Behavioural ecology, Ecology, Zoology, Animal behaviour

## Abstract

The study of non-human multilevel societies can give us insights into how group-level relationships function and are maintained in a social system, but their mechanisms are still poorly understood. The aim of this study was to apply spatial association data obtained from drones to verify the presence of a multilevel structure in a feral horse society. We took aerial photos of individuals that appeared in pre-fixed areas and collected positional data. The threshold distance of the association was defined based on the distribution pattern of the inter-individual distance. The association rates of individuals showed bimodality, suggesting the presence of small social organizations or “units”. Inter-unit distances were significantly smaller than those in randomly replaced data, which showed that units associate to form a higher-level social organization or “herd”. Moreover, this herd had a structure where large mixed-sex units were more likely to occupy the center than small mixed-sex units and all-male-units, which were instead on the periphery. These three pieces of evidence regarding the existence of units, unit association, and stable positioning among units strongly indicated a multilevel structure in horse society. The present study contributes to understanding the functions and mechanisms of multilevel societies through comparisons with other social indices and models as well as cross-species comparisons in future studies.

## Introduction

A multilevel society is a social structure with nested levels of social organization. Individuals are structured in stable unit groups that preferentially associate with other units to form a higher level of social organization^1–5^. Humans, for example, live in a multilevel society where families gather to form a local community, families further combine to form higher social organization levels such as suburbs, cities, states, and countries. As a multilevel society is characterized by polyadic interactions among units, it is important to understand how such group-level relationships have evolved and been maintained. Multilevel societies occur in various taxonomic groups of mammals, such as primates^6,7^, cetaceans^8^ and equines^9–11^, and have recently been found in one avian species^4^. Previous studies have revealed that multilevel societies occurred sporadically via different evolutionary processes, resulting in a large structural variety among various taxa^12^. The association pattern is different at each social level in each species, even if the name given to that level of social organization is the same^2,5,7,12,13^. For example, the third-level organization of hamadryas baboons *(Papio hamadryas),* i.e. “band”, shows consistent association throughout a year^12^, while the third-level organization of sperm whales (*Physeter macrocephalus*), i.e. “clan” (as a side note, “clan” refers to the second-level group in hamadryas baboons), is much more unstable, and often lasts for a few days^8^. This makes it difficult to compare species and populations^7^.

In order to gain a better understanding of multilevel societies, quantitative evaluations at different organizational levels and detailed comparisons among different species and populations are necessary^2^. Recently, an increasing number of studies have applied numerical methods to evaluate the association patterns and social structures of a multilevel society. The most frequently used index for quantifying social relationships is the association index (AI)^7,9,14–18^. It measures the portion of time individuals spend together in every dyad. Clustering techniques or community detection algorithms are applied based on the AI scores to detect the stratification of associations. It is concerning that some studies have arbitrarily decided the distance that suggests that two individuals are “together” without examining whether it actually implies intimacy of the animals (c.f. ^9,17,19^, but see ^18^). If the threshold distance is too small, it may miss the interactions at higher levels of social organization, while if it is too large, it may not be able to detect the units. This causes difficulty when conducting a meta-analysis because each study determines the threshold individually, so the investigation of associations occurs at different resolutions.

**Figure 1:**
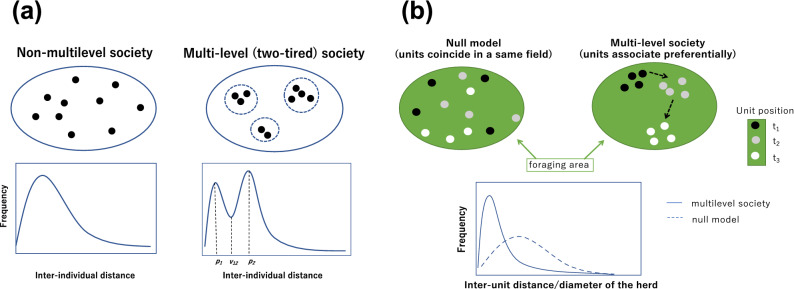
(**a**) The prediction on the histogram of inter-individual distances. When the group contains more than two-levels of social organization with different sets of parameters for the attraction–repulsion model, the histogram should be multi-modal. The distance of the first peak and the second peak are referred to as *p*_1_ and *p*_2_, and the valley between *p*_1_ and *p*_2_ is *v*_12_*. p*_1*,*_ and *p*_2_ could be considered as the most frequent value of inter-individual distances within a unit and between units, respectively. *v*_12_ represents the threshold that divides the intra- and inter-unit association. (**b**) The prediction on the inter-unit association. If units just coincide in the same field (null model), units just spread in the foraging area. However, in multilevel society, units preferentially associate and forage together. Thus, the inter-unit distance in a multilevel society should be smaller and have steeper distribution than that of null model. The graphs were created using Microsoft Power Point for Mac 16.16.25 (https://office.live.com/start/powerpoint.aspx).

The aim of the current study was to use actual distances between individuals to develop more rigorous methods for detecting a multilevel society and to evaluate their association patterns. The spatial aggregation of animal groups is often explained using a repulsion and attraction model^20,21^. According to the model, animals are likely to be within a certain distance from their neighbours when a kinetic equilibrium occurs. In a multilevel society, animals should know the unit to which they belong and identify their own unit members, while also allowing other units to stay together at a higher-level^22,23^. We assumed that different levels of social organizations had different levels of equilibrium. In other words, when animal groups consist of two or more levels of social organization, a histogram of inter-individual distances should be multi-modal (c.f. ^24^). In the case of a two-level multilevel society, the most frequent values of inter-individual distances are within a unit and between units, respectively (Fig. 1a). Units could be identified using an association index with a threshold around the first peak because the same unit members should maintain the distance around it, while those from different units are likely to avoid approaching that close.

In addition, each peak of the histogram should be centred around a lower value than the null-model when animals are in fact gathering, not just when they coincide in the same place without any social drivers (i.e., null model/random associations among units; Fig. 1b)^25^. It is highly likely that units have heterogeneous association patterns and positional tendencies when units have non-random social relationships. When units prefer or avoid certain units, such units would maintain a smaller or greater distance compared to the other units, which would result in greater variations in association rates compared to the null model. The dominance hierarchy can also affect the spatial positioning among units because at an individual level, it is known that dominant individuals tend to occupy the centre of a group in various taxa^26^. Such positional and association patterns can also reinforce the prediction of unit social aggregation, which would help understand the function of the multilevel society.

Equines are one taxon that exhibits a multilevel society. Most equine studies have focused on plains zebras (*Equus quagga*)^9^, but multilevel societies have also been reported for the Przewalski’s horse (*Equus ferus przewalskii*)^11^, one subspecies of Asian wild ass (*Equus hemionus luteus*)^10^, and one population of feral horses (*Equus caballus*)^27,28^. The societies of these species are two-tiered. In the current paper, we refer to the lowest level of organization as a “unit” and the association of units as a “herd”. Units contain two types of social organization: a harem composed of one or two adult males, several females and immature individuals, or an all-male unit (AMU) or bachelor group, which is composed of adult males that could not attract any females^29^. Unlike harems, whose memberships are usually stable for a few years or more^30^, herds show a fission–fusion structure and can sometimes contain hundreds of individuals. According to studies on feral horses in Wyoming’s Red Desert^27,28^, harems that share a foraging area follow similar seasonal movements and have a hierarchical relationship when using a waterhole; thus, it can be argued that those harems form a herd. This synchronization of seasonal migration is also observed in studies of other feral populations^13^, indicating that herd formation is rather common, and not specific to Wyoming’s Red Desert. However, the presence of multilevel societies in feral horses is still questionable, as no study has examined whether the units gather to form a social organization or if they are simply together because of the abundance of resources^13^. To verify the presence of the herd, comparison with a null model that takes into account the effect of the environment is necessary.

Using distance metrics requires accurate spatial positioning of a focal group’s members. The recent development of remote sensing techniques using unmanned aerial vehicles (UAVs, drones) enabled us to acquire spatial patterns and record movements of large groups more easily and accurately than before. Feral horses show great potential for studying multilevel societies. They live in an open habitat and there are many populations habituated to humans, which makes them a perfect research subject to test this new methodology using drones. In addition, horses have the potential for cross-population and cross-species comparisons, as feral populations exist worldwide. Our team has already applied these drone techniques for the observation of feral horses living in Serra D’Arga, a mountain located in the north of Portugal^31^, successfully identified individuals at an intra-harem level, and analysed their positioning patterns^32–35^. During the breeding season, multiple harems and bachelors gather in the same field site and seem to form a large structure (i.e., a herd) without losing unit structure, suggesting the presence of a multilevel society in the feral horses. In this study, we further developed an identification method for a large number of horses to investigate how spatial data can be used to define and evaluate the horse society in Portugal.

Our study tested whether the horse society had a multilevel structure using positional data in three steps: (1) we examined them for the presence of unit groups, (2) we tested whether units were aggregating to form a herd, and (3) we determined whether units have stable positional patterns within the herd by using social network analysis. We hypothesized that (1) the distribution of inter-individual distances should be multi-modal if units exist, (2) observed inter-unit distances should be smaller than randomized data, and (3) association rates and centrality are significantly different among units.

## Results

### Presence of units

**Figure 2:**
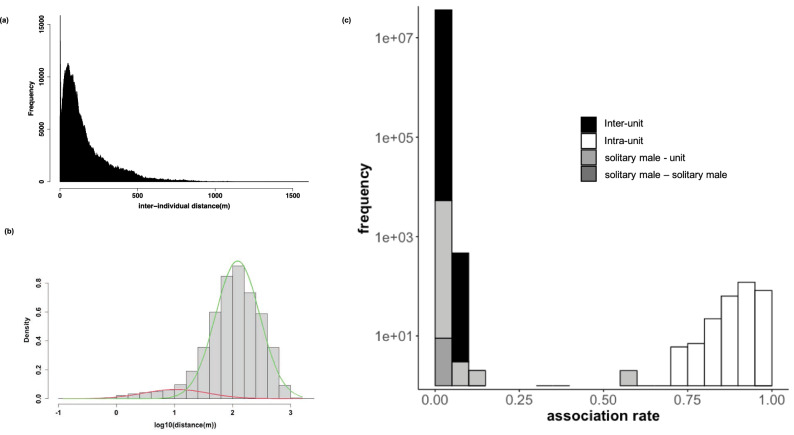
(**a**) Histogram of inter-individual distances showing clear bimodality. (**b**) The inter-individual distance was converted to logarithmic scale and then fitted to Gaussian mixture model. The green and the red lines represent the two estimated Gaussian distributions. (**c**) The unit-level association rate also showed bimodal structure. We later examined the histogram of (**c**) to investigate how the association pattern was distributed according to the individual’s belonging. The y axis of (**c**) is on a logarithmic scale. The graphs were created using the statistical software R^49^.

The histogram of the inter-individual distances showed two peaks (Fig. 2a). The Expectation–Maximization (EM) algorithm^36^ fitted the inter-individual distance distribution to the Gaussian mixture model (Fig. 2b). The estimated parameters were (${w}_{i},{\mu }_{i}, {\sigma }_{i}$) = (0.0879, 1.0212, 0.5560) and (0.9121, 2.0866, 0.3819). The two peaks were *p*_*1*_ = 10^1.0212^ = 10.5 m and *p*_*2*_ = 10^2.0866^ = 122.1 m. These two Gaussian distributions intersected at x = 1.18921. Thus, *v*_*12*_ was defined as 10^1.18921^ = 15.5 m. We used this distance as the threshold that separated intra- and inter-unit associations in the following analysis. The histogram of the intra-unit-level association rate (the ratio of the observation that each dyad was observed closer than *v*_*12*_) also showed a clear bimodal structure (Fig. 2c). There was a large gap in the intra-unit-level association around 0.15–0.70. We considered dyads with intra-unit-level association rates larger than 0.70 as the same unit members, given that one member of the pair was seen.

Our analysis identified 23 units (21 harems and 2 AMUs), and 5 solitary males. The mean ± SD of the intra-unit-level associations were 0.91 ± 0.05 between individuals in the same unit and 0.009 ± 0.013 between those in different units. The mean ± SD distance of nearest-individuals within and between units were 3.2 ± 7.7 and 39.3 ± 56.1 m, respectively, and the mean ± SD inter-individual distances within and between units were 6.9 ± 7.5 and 170.5 ± 157.1 m, respectively.

This perfectly matched with the division we made based on manual group partitioning from the ground observations. One male, Hirosaki, had one connection (association rate: 1.0) with one of the males in a harem with two males, Takaoka and Uozu, but not with other members (0.57–0.69). Thus, we categorized this male as solitary. In fact, we often observed fighting between Uozu and Hirosaki, which rarely occurred between Uozu and Takaoka, and males in other multi-male harems. From this, we presumed that Hirosaki stalked the harem to gain females. Among the other four solitary males, two were completely solitary as they moved within 15.5 m of other individuals only once and twice, respectively. The other two showed flexible associations (they occasionally were alone farther from the other horses, they sometimes followed AMUs and at other times they were stalking a harem). The size of the harems ranged from 2 to 9 horses (average 5.3): 1–2 adult males, 1–7 adult females, and 1–2 young individuals (Supplementary Table S1). Four harems were multi-male and the other 17 were single-male. Horses in the same unit always appeared in the field site together, with a single exception that a female named Machida from Harajuku group, did not appear in the field on June 20th, although the other unit members did.

### Association of units

**Figure 3:**
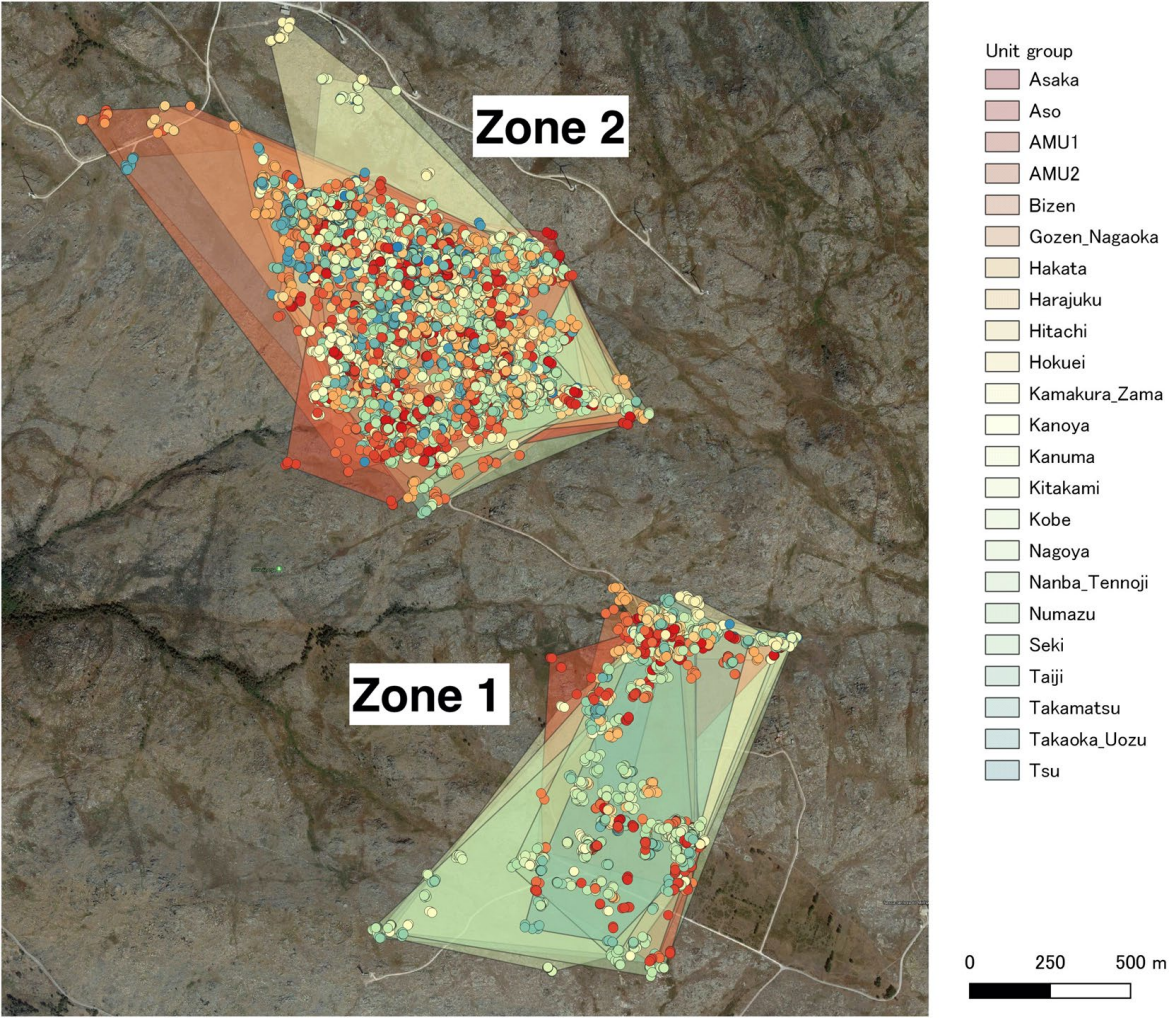
Observed horse position and the convex hulls that surrounds all of the observed positions of each unit. Each unit has different colour dots and convex hulls. The image was created under QGIS 3.6 environment (https://qgis.org/ja/site/index.html).

A total of 17.9 ± 4 harems (85.2 ± 19.2% of 21 harems) and 1.5 ± 0.5 AMUs (75.0 ± 25.6% of 2 AMUs) were observed at the observation site each day on average (see Supplementary Fig. S4 for more detailed daily availability). UDOI was 1.10 ± 0.34 (mean ± SD); UDOI > 1 indicates that two home ranges are associated with a high degree of overlap^37^. Each unit had 13.21 ± 5.1 others (out of 22 units) whose UDOI was larger than 1. Until July 4th, the units foraged in Zone 2 and the horses were rarely observed in Zone 1. However, from July 5th to 10th, horses were also observed in Zone 1 and sometimes split between Zone 1 and Zone 2 (Fig. 3). The association networks of these two periods had a high correlation, with r = 0.99 (Mantel test, *p* < 0.001; Supplementary Appendix). In addition, the distance to the nearest unit was significantly smaller until July 4th (mean ± SD: 35.0 ± 32.3 m) than after (54.4 ± 100.8 m; t (980.53) = 5.77, *p* < 0.001) according to Welch’s test (Supplementary Appendix).

**Figure 4:**
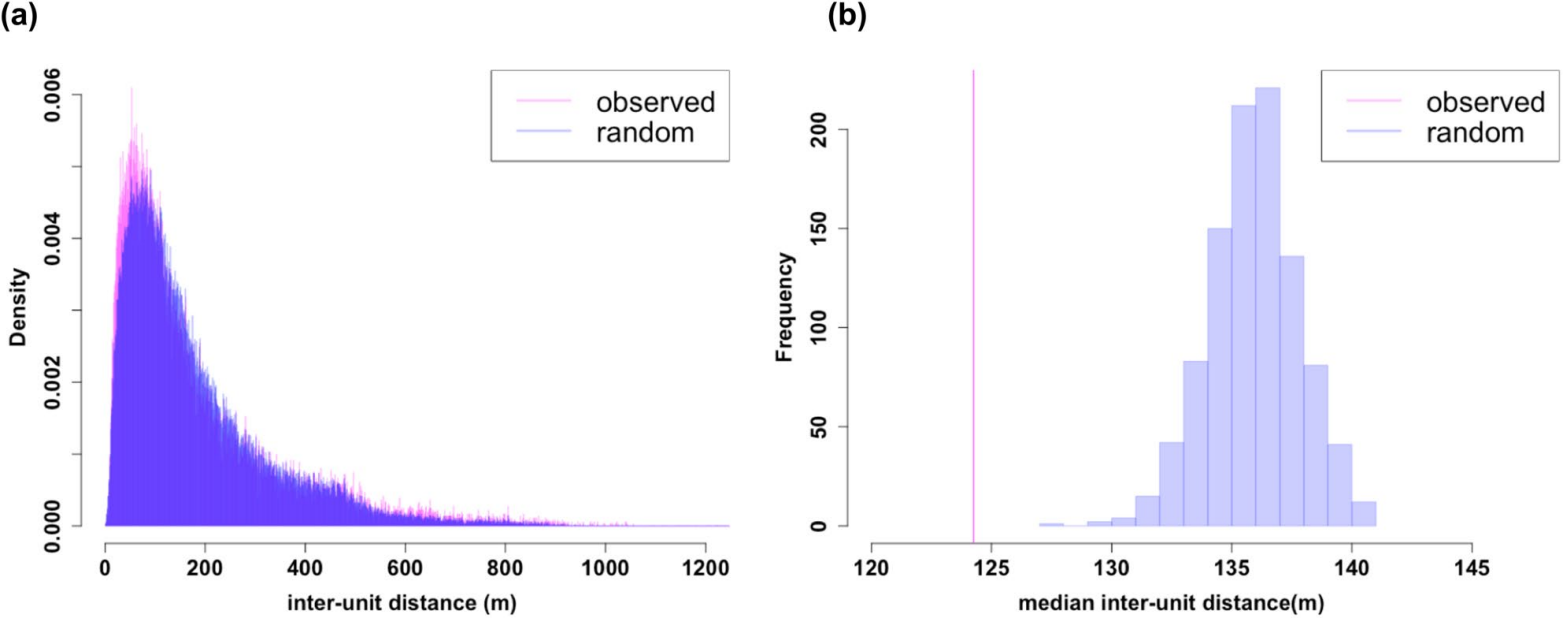
Histogram of (**a**) interunit distances and (**b**) the maximum interunit distances at each observation time and Zone. The graphs were created using the statistical software R^49^.

The permuted inter-unit distance and observed distance differed significantly according to the Kolmogorov–Smirnov test (*p* < 0.001, Fig. 4a). Observed median inter-unit distances (median [1st, 3rd quantile] = 124.3 [66.9, 240.2] m) were significantly smaller than that of any randomized data (*p* < 0.0001, Fig. 4b).

### Unit-level social network analysis

**Figure 5:**
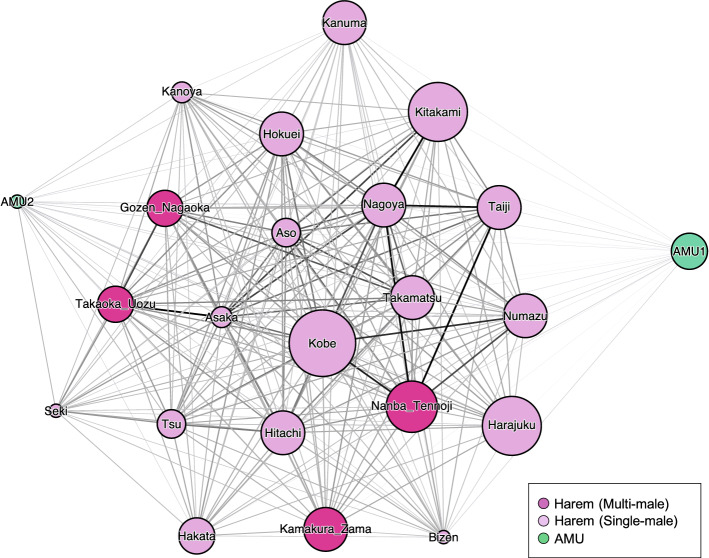
The social network based on the association matrix. The edge thickness represents the value of the simple ratio index. It was created with ForceAtlas algorithm under Gephi0.92 environment^63^. Nodes represent units, where the pink one represents a multi-male harem, the light pink represents a single-male harem, and the green represents an AMU. The names of the harems were based on the names of the adult male/s.

The network of inter-unit association was shown in Fig. 5. In unit-level social network analysis, we assumed that two units were preferentially associated when they were within *p*_*2*_. The networks based on the average inter-unit distance had a significant positive correlation with this network (r = 0.78, *p* < 0.001) according to the Mantel test.

The results of the regression analysis on the unit-level network are shown in Tables 1, 2 and 3. The association rate among units correlated with the unit type similarity (coefficient: − 0.0685, p (|β| ≦ |r|) = 0.0001), but not with the unit size similarity (coefficient: − 0.0045, p(|β| ≦ |r|) = 0.061; Table 1). Harems showed significantly larger strength centrality (mean ± SD: 17.1 ± 1.8) than AMUs (mean ± SD: 9.9 ± 2.5, coefficient ± SE: 4.90 ± 1.07, t = 4.57, *p* = 0.0012; Table 2). Unit size positively correlated with strength centrality (coefficient ± SE: 0.340 ± 0.138, t = 2.46, *p* = 0.023), but the number of harem males had no significant correlation (t = 0.89, *p* = 0.40; Table 3).

**Table 1:** The result of MRQAP-DSP (10,000 permutation). |size_i_ − size_j_| and |centrality_i_ − centrality_j_| is differences in unit size and centrality between units, type similarity is 0 when two units were same type (AMU or harem) and 1 when not, and location overlap is the proportion of the observation when two units were observed in the same zone.

	Partial correlation	p(β≧r)	p(β≦r)	p(|β|≦|r|)
(Intercept)	0.07088396	0.9951	0.0049	0.0087
|Size_i_ − size_j_|	− 0.0045139	0.0308	0.9692	0.061
Type similarity	− 0.0685101	0.0001	0.9999	0.0001
|centrality_i_ − centrality_j_|	− 0.0029437	0.1027	0.8973	0.1988
Location overlap	0.51052251	>0.9999	<0.0001	<0.0001

**Table 2:** The result of regression analysis on strength centrality of harems and AMUs. p ( <|t|) is based on the t value as a normal consequence of linear regression without permutation and p (perm) is calculated based on the permutation.

	Strength centrality				
	Coefficient	SE	t	p ( <|t|)	p (perm)
(Intercept)	7.1120	2.0707	3.4346	0.00262	NA
Harem ( +) / AMU( −)	4.9012	1.0733	4.5665	0.00018	0.0012
Observation no	1.4179	0.3092	4.5855	0.00017	0.0002

**Table 3:** The result of the regression analysis on the strength centrality of harems. p ( <|t|) is based on the t value as a normal consequence of linear regression without permutation and p (perm) is calculated based on the permutation.

	Strength centrality				
	Coefficient	SE	t	p ( <|t|)	p (perm)
(Intercept)	14.20241	1.515436	9.3718	3.97E − 08	NA
Harem size	0.34072	0.138389	2.4620	2.48E − 02	0.02279772
Stallion no	0.59500	0.671318	0.8863	3.88E − 01	0.40035996
Observation no	1.31685	0.269047	4.8945	1.37E − 04	0.00039996

## Discussion

Our attempt to apply drone technology to acquire vast numbers of identified individuals’ positions was successful, with more than one hundred individual feral horses identified from aerial photos. Although drones have an advantage in recording the spatial position of large groups, individual identification has only been attempted in few studies and only with a small-sized group^32^. Thus, most drone studies have focused on temporary social interactions and collective behaviour in behavioural ecology (cf. ^11,38^). Our study indicated a further potential of drones for the long-term monitoring of social associations by adding individual information on positional data.

As a result of analysing positional data, this study provides strong evidence of a multilevel structure in feral horse society with: (1) the presence of units, (2) association of units to form a higher-level social organization, the herd, during the observation period, and (3) the stable pattern of unit positioning.

Firstly, the histogram of inter-individual distances showed clear bimodality, suggesting the presence of smaller groups (harems and AMUs). The two peaks roughly matched the average distances of the nearest individual within and between units, and members in each unit stayed within the threshold distance (*v*_12_) for most of the time, while members between different units rarely came closer than the threshold. These results support our assumption that the first peak represents the inter-individual distances within units, and the second peak represents those between units. Although this method worked well overall, it does not allow for the composition of units to change during the observation period. We observed some solitary bachelor males with unstable social relationships as they often changed the individuals they accompanied. We should reconsider how to evaluate such temporal changes in grouping in future studies.

Secondly, further analysis showed that the assembly of units formed a herd not just because units occupied the same location. Their foraging areas largely overlapped; thus, they did not have a territory. Moreover, the observed inter-unit distances were smaller than the randomized data, which suggested attraction among units. We observed an increase in the nearest-unit distance after units started to forage in Zone 1 from June 5th, which may be because units were starting to disperse, losing the herd structure at the end of the breeding season. In addition, the network analysis suggested that units were not just randomly associated with others, or that they were associated with certain units. The regression analysis showed a correlation between unit type similarity and the association rate, although the effect size was not very large. As we started our observations from 2016, we still did not know about their detailed genealogy and life history (e.g., where they were born and how they transferred from the units that they belonged to), which is highly likely to have considerable effects on inter-unit social relationships^9^. A long-term accumulation of observation would be needed to further investigate the factors that determine the inter-unit association patterns.

Finally, network analysis revealed the spatial structure of the herd, where larger harems occupied the centre and AMUs tended to be at the periphery of the herd. In many social animals, dominant individuals often occupy the centre, forcing subordinates to the periphery^26^. Applying the dominant-centre rule to group-level social relationships, our data suggests that the hierarchical relationship is between units and correlates with harem size. For many group-living animals, including social insects, wolves, hyenas, lions, humans, and other primates ^39^, the ability to infer social dominance by assessing the numerical size of one’s own group relative to another has evolved to reduce aggressiveness between groups. It has also been reported that larger feral horse harems have priority access to water resources ^28,40^. However, inter-unit hierarchy has rarely been reported in multilevel societies (but see^11,15,41,42^) because of the difficulty in measuring it due to few aggressive behaviours among units ^43^. Spatial positioning could be a good parameter for measuring the dominance rank among units.

AMUs were often found to be at the most peripheral zone of the herd. Bachelor’s threat hypothesis argues that harems assemble to form coalitions to decrease the risk of harassment, especially infanticide, and harem takeover by bachelors, which is presumed to be the most plausible scenario for multilevel society evolution in plains zebras^44^ and Asian colobines^45^. Infanticide has been witnessed in both feral and captive horses, and foreign males are involved in most cases^46,47^. Our previous study by Inoue et al. (2019)^32^ found that harem males tended to locate themselves in the outer area of units. This is supposedly because harem males are protecting their females and foals from bachelors. Our discoveries on the positional differences between harems and AMUs among units, and females and males among harem members support the assumption that herd formation benefits harems via more effective protection from bachelors. This is also consistent with the fact that this association of the units occurs only during the breeding/birth season.

These three results strongly indicate a multilevel structure in feral horse society. Our method of investigating multimodal inter-individual distance distributions and the subsequent null-model analysis could be applied to other populations and taxonomic groups to detect modular group structures with a minimum arbitrary definition.

It has been reported that the association pattern of multilevel society changes dynamically^10,18^ and differs among species^1,17^ because of various environmental and social factors. Adding information on group spatial structure may facilitate further understanding of the functions and mechanisms underlying the formation of multilevel social groups. For example, if herd formation benefits harems by providing effective protection from bachelors, it is highly probable that the preference and cohesiveness of harems toward the centre of the herd may change in response to the number of bachelors nearby. These spatial data could be used as an index to conduct cross-species comparisons. For example, comparing our data with the study by Ozogany and Vicsek^11^ showed that units of the Przewalski's horse appeared much more aggregated than those of the feral horse. Their study suggested the pyramid-like structure of a herd, where the leader harem initiates the movement from the front. We have never noticed such behaviour in the feral horse population we observed. Although the social structure and reproductive strategy of these two species are quite similar^13,30^, the spatial dynamics of their herds seemed considerably different. Further investigation of the spatial structure of various multilevel societies may reveal new aspects to us.

In conclusion, our study described an innovative methodology that enabled the quantitative definition and evaluation of multilevel societies using drones. More long-term observations, including collecting other social indices such as genetic relatedness and social interactions (e.g., fighting), are needed to investigate the repeatability of the method and to fully understand the dynamics of feral horse group structure, since this study only dealt with spatial data over a short period of time. Interpopulation and interspecific comparisons of multilevel societies have never been conducted with a non-primate species^2^. If our method is applied to other species, especially other equines, including feral horse populations, it would enable a meta-analysis based on inter-individual distance distributions and network metrics, which may cast new light on multilevel societies. Further studies on horses and other species are necessary to optimize this method and to explore a way to conduct cross-population and cross-species comparisons.

## Methods

### Data collection

We conducted observations from June 6 to July 10, 2018 in Serra D’Arga Portugal, where approximately 200 feral horses were living without human care^31^ (see Supplementary Appendix for detailed information of the site). The observation period corresponded with the breeding and birth season of the horses. The field site had two large flat areas, Zone 1 and 2, which were separated by rocky hills. We used drones (Mavic Pro: DJI, China) to measure accurate distances between all individuals in the observation area. The flights were performed under clear sky conditions at an altitude of 30–50 m from the ground, and we took successive aerial photographs of the horses present at the site at 30-min intervals from 9:00–18:00. The average duration of each flight was 4 min 27 s ± 3 min 5 s. As we had been using drones on this site since 2016^31,32^, the horses were acclimated to the drones and did not show any behavioural response to UAV operations at this distance (see Supplementary Appendix for a more detailed explanation of the drone operation). All procedures performed in the studies followed international, national, and institutional guidelines for the care and use of animals. The field observations complied with the guidelines for animal studies in the wild issued by the Wildlife Research Center of Kyoto University, Japan (https://www.wrc.kyoto-u.ac.jp/guidelines/wild.html; in Japanese), and we signed a Memorandum of Understanding with the Viana Do Castelo municipality, which governs the study area and our field station. No further formal permission was required prior to conducting our research.

**Figure 6:**
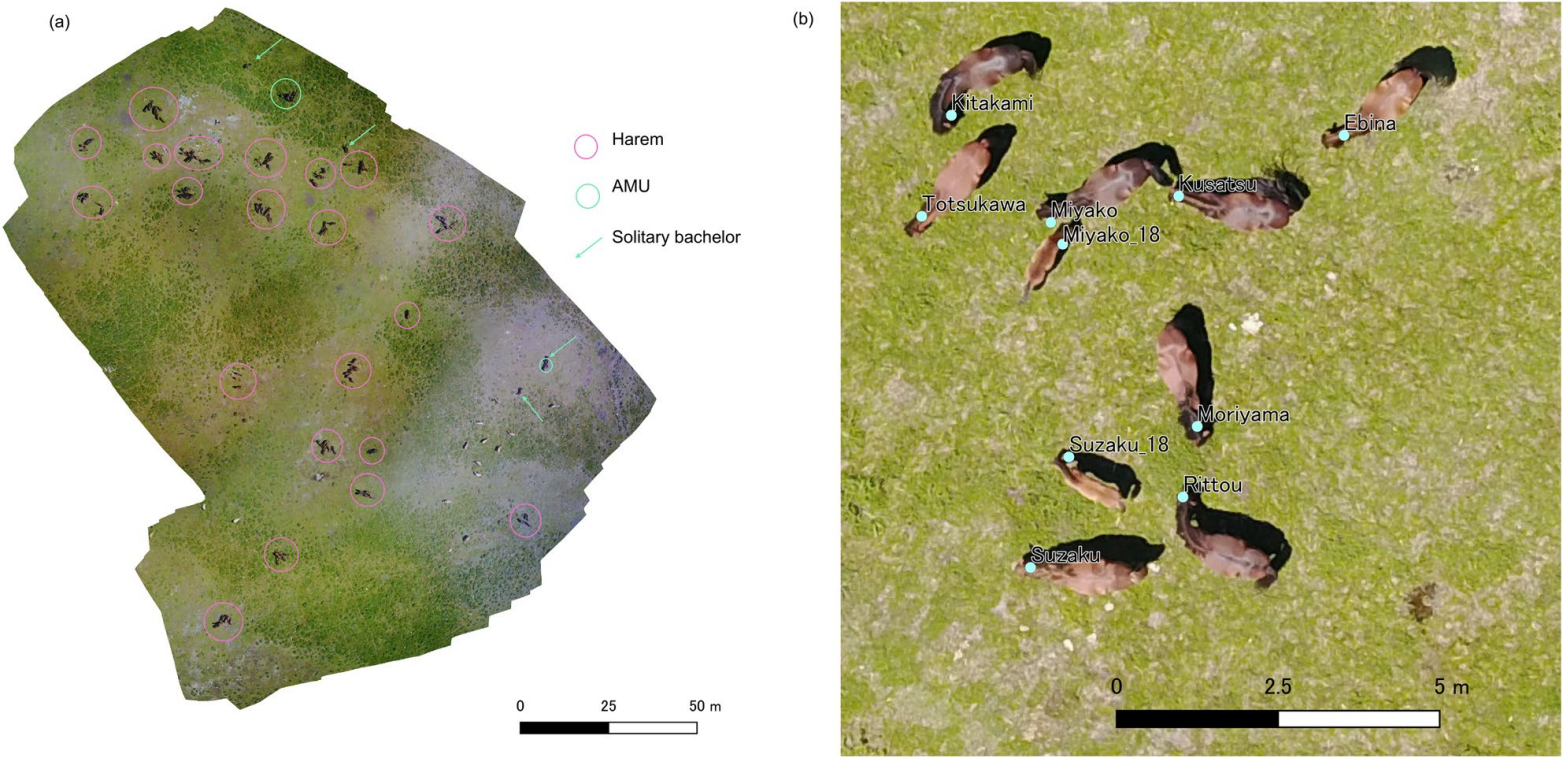
(**a**) An example of the orthomosaic created from the successive aerial images from a drone. Each brown dot on the field is a horse. Circles and arrows represent the units and solitary males partitioned based on the distance distribution (see the results for details). White dots are free-ranging cows. (**b**) The enlarged view of an orthomosaic. It captured an upright image of a harem named Kitakami. All horses on the orthomosaics were identified. The points and labels represent the positions and IDs of each horse. Infants were named as “mother’s name”_18. Both of the images were created using AgiSoft Photoscan professional 1.4.3 (https://www.agisoft.com).

Orthomosaic imaging was conducted using AgiSoft PhotoScan Professional software version 1.4.3 (currently referred to as AgiSoft Metashape). The software connected successive photos and created orthophotographs in the GeoTIFF format under the WGS 84 geographic coordinate system. We first identified all horses from the ground and made an identification sheet for all individuals, recording their physical characteristics such as colour, body shape, and white marks on the face and feet. All horses in the orthophotographs were identified accordingly. We estimated their age class as either adult, young, or infant. The adults were individuals who experienced dispersal from their natal group, the young were those who were born in or before 2017 and still belonged to their natal group, and the infants were individuals born in 2018. We excluded infants from subsequent analyses because their position seemed highly dependent on their mothers. An orthomosaic corresponds to the coordinate system at a pixel level; thus, it can be handled as a raster data structure in a QGIS environment. We positioned the heads of the horses, and all locations were stored in shapefile formats (Fig. 6). Horses sometimes rested under trees and were not visible from drones. In these cases, we identified the horses from the ground using binoculars and recorded the position of the tree. We did not record the inter-individual distance in this case, but when we calculated the average position of the units, we used the centre of the tree as a representative of their positions. We considered that the horses under the same tree were associated at the unit-level while calculating the intra-unit-level association rate because the diameter of the trees was less than *v*_*12*_ (15.5 m). When there were other horses closer than 15.5 m from the centre of the tree, we also counted them as associated. In addition, we sometimes missed recording the horses. We eliminated the data with more than 30% of the available horses located outside of the orthomosaic.

The coordinate system was converted to a rectangular plain WGS 84 / UTM Zone 29 N, and the distances between all pairs of individuals in the same zone were calculated. In total, 238 orthomosaics were obtained in 19 days, and 21,445 non-infant individual positions were obtained. A total of 126 non-infant horses (119 adults: 82 females and 37 males, 7 young individuals: 6 females and 1 male) and 19 infants (11 females and 8 males) were successfully identified. One adult female, named Oyama from Kanuma harem, disappeared sometime between the evening of June 15th and morning of June 16th, probably predated by wolves. We observed 97 ± 28 individuals (mean ± SD: 77.0 ± 22.3%) in each observation, and we recorded 90 ± 28 individual locations (71.5 ± 22.3%). We found that 473 horses had become invisible to drones and 1172 available horses were located outside of the orthomosaic in total. In each observation, 92.8 ± 9.1% of the horses in the observation site were successfully recorded. We considered each observation to be independent because at the unit-level, 8 min were enough for them to change the association^48^, and horses moved 44.1 [30.5, 93.7] m (median [1st, 3rd quartile]) in 30 min, which should be enough to change unit-level association as well.

### Investigation into the presence of units and their definition

The rest of the analysis was performed using the statistical software R 4.0.0^49^. We first examined whether the histogram of inter-individual distances was single- or multi-modal. We referred to the distance at the first and second peaks as *p*_1_ and *p*_*2*_, respectively, and the distance at the valley between the first and second peaks as *v*_*12*_ (Fig. 1a). We referred to associations less than *v*_*12*_ as intra-unit-level association specifically.

We fitted the inter-individual distance distribution to Gaussian mixture modelling with the Expectation–Maximization algorithm using the R package ‘mixtools’^36^. The distance data was transformed to a natural logarithmic scale and then fitted to a Gaussian mixture model, i.e., ${\sum }_{i=1}^{k}{w}_{k}\times N({\mu }_{k}, {\sigma }_{k}^{2})$, where k is the number of components. We compared the Bayesian Information Criterion (BIC) of k = 1, 2, …, 20. BIC almost converged to the same value when k ≧ 2, therefore, we decided to use k as 2 (Supplementary Fig. S5). We defined *p*_1_ and *p*_2_ as ${10}^{{\mu }_{1}}$ and ${10}^{{\mu }_{2}}$ and log_10_ (*v*_*12*_) as the intersection values of the two Gaussian functions. A network was generated from an association matrix. We first set *v*_*12*_ as the threshold of intra-unit level association. Horses from the same unit are likely to keep distance smaller than the threshold, and those from different units avoid getting closer than that threshold. Thus, we also examined whether the intra-unit-level association rate also showed a bimodal structure. We calculated the association rate using a simple ratio index (SRI, the probability of observing both individuals together given that one has been seen). We chose SRI as an index according to the recommendation of Hoppit and Farine^50^.

We examined each unit to ensure that individuals were connected to all the other unit members. If not, we excluded those individuals from the unit and considered them as solitary. Some solitary males were also detected, but we did not count them as units because they did not regularly show up in the observation sites, and even when they appeared (Supplementary Fig. S4), they were mostly located in the peripheral area or they followed a certain harem or AMU. To test the validity of this method, we also manually grouped individuals into groups, where two observers decided units intuitively based on direct observations from the ground. We compared this with partitions based on distance data.

### Investigation of inter-unit association

The bimodal histogram of association rates may occur even without any multilevel social structure; for example, when each unit has its own territories or they overlap in a site without any social interaction. To exclude these alternatives, we examined whether their foraging area during observation periods overlapped and whether the inter-unit distances were smaller than randomized data (Fig. 1b).

Firstly, we examined the home range overlaps of the units. We determined the central position of a unit as the mean position of non-infant members, and the utilization distribution overlap index (UDOI)^37^ was calculated for all pairs of units using the R package ‘adehabitatHR’^51^.

Secondly, we defined inter-unit distance as the distance between the center of the units. We then conducted a randomization to test if the observed inter-unit distance was smaller than the randomized data. We only shifted the observation time of horses, so that the dates of observation and the trajectories of horses were maintained. This constraint eliminates the possibility that the spatial selectivity of horses drove the unit aggregation without invoking any social mechanisms and to maintain the natural movements of horses. Observation time was randomly assigned from three choices -30, 0, or + 30 min for each unit daily. The permutation was conducted for 1000 repetitions for all data. The observed median inter-unit distances were compared to those calculated from permuted datasets.

### Creating unit-level social network

We conducted a social network analysis to reveal the association pattern and spatial structure of the multi-unit group. Social interactions of the horses were likely to occur mainly with individuals in close proximity. The global structure of a group emerges from local interactions in many animals^52–54^. Thus, we rearranged the distance data to association data by applying a method similar to that used for defining units.

Networks were generated for each sampling period (i.e., each flight of drones), where nodes were defined as each unit, and edges were scaled to 1 when the inter-unit distance was smaller than *p*_2_ (the second peak; Fig. 1a), or otherwise scaled to 0 (the validity of this threshold value was examined in Supplementary Appendix). When a pair of units were connected with each other either directly or indirectly via another unit, they were considered to be associated. Solitary bachelors were excluded from the analysis. A unit-level association matrix was created from this co-membership data using the SRI^55^.

We assumed that the network represented the actual spatial structure of the units. To confirm our assumption, we compared it with a network created from the average distance matrix between all dyads of individuals. The distance matrix was converted to an association matrix by taking the inverse of the squared average inter-individual distance. We tested the correlation between these two networks by a Mantel test with 9999 randomizations using the R package ‘ade4′^56^.

### Social network analysis based on permutation

For the network based on the association (e.g., inter-individual distance), it has been recommended to use data stream (pre-network) permutation, which swaps pairs of nodes to obtain randomized networks from shuffled group affiliations^57^. The advantage of the data stream permutation compared to post-network analysis is that it can control the confounding effects, for example, sampling bias and location preferences, by constraining swaps to occur only within the same time period or location^57,58^. However, a recent study pointed out that data stream permutation could cause high type I (false positive) error in regression analysis when the society structure is non-random because the permutation procedure could decrease the variance of the network variables^59^.

We first carried out data-stream permutations using the R package ‘ANTs’^60^. In total, 10,000 random networks were created. The swaps were constrained by the sampling period and zones to control the units entering and leaving the observation sites. The edge weight of the observed network was 0.375 ± 0.099 (mean ± SD). The SD of the network was significantly higher than that of the permuted network (p_right_ =  0.003).

Franks et al.^58^ provided a possible solution to control the confounding effects using post-network analysis by importing their covariates into the regression analysis. For example, they added the location overlap into the model as a covariate in dyadic analysis because associations could be affected by the preferences for the locations. In the analysis of strength centrality, the sum of weights attached to edges belonging to a node^61^, they recommended placing variables representing the sampling bias because oversampling may cause the excess value of strength .

We conducted a regression analysis with the DSP procedure following the method explained by Franks et al.^58^. We first developed a model to investigate whether the associations (edge weights) of each dyad could be explained by coincidence in unit type (AMU or harem) and similarity in unit size. To eliminate the effect of location preferences, we used the ratio of two units that appeared in the same field on the same day. On a smaller scale, the unit positional centrality could also affect the association; for example, the units in the central position are more likely to be closer to the units in the center, rather than those in the periphery, as a natural consequence. Our analysis revealed that the strength centrality correlated with the positional centrality (see Supplementary Appendix), so we also added the difference in the strength as a covariate in the model. Multiple regression quadratic assignment procedures (MRQAP) with DSP with 10,000 permutations were performed with ‘asnipe’ package in R^62^.

The other model was built to investigate how strength centrality differed between harems and AMUs and according to the harem characteristics, i.e., size and stallion number (either 1 or 2). We also included the mean-centred number of observations as a covariate to control for sampling bias. We used the R code provided by Franks et al.^58^ and conducted the DSP with 10,000 permutations of the residuals. The significance level was set at 0.05 for all tests.

## Supplementary information

Supplementary Information 1.
