# Supplementary material for: Aerial drone observations identified a multilevel society in feral horses
Source: Sci Rep. 2021 Jan 8;11:71. doi: 10.1038/s41598-020-79790-1 (PMC7794487; doi:10.1038/s41598-020-79790-1)
Supplement: Supplementary file 1 — Supplementary Information 1. [file 41598_2020_79790_MOESM1_ESM.pdf]

Aerial drone observations identified a multi-level society in feral horse

Tamao Maeda[1,*], Sakiho Ochi[1], Monamie Ringhofer[2], Sebastian Sosa[3], Cédric Sueur[3,4], Satoshi Hirata[1] and Shinya Yamamoto[2,*]

[1]Wildlife Research Centre, Kyoto University, 2-24 Tanaka-Sekiden-cho, Sakyo, Kyoto, Japan

[2]Institute for Advanced Study, Kyoto University, Yoshida Ushinomiya-cho, Sakyo, Kyoto, Japan

[3]Université de Strasbourg, CNRS, IPHC, UMR 7178, Strasbourg, France

[4] Institut Universitaire de France, Paris, France

* tamao@powarch.com, shinyayamamoto1981@gmail.com

Supplementary Appendix

Study area

The study site is in Serra D'Arga, a mountain located in northern Portugal (Figure S1a, 8°420W, 41°480N), whose highest peak is 825 m. Serra D'Arga is in a cool summer Mediterranean climate according to the Koppen-Geiger classification system. The higher area of the mountain is rocky but abundant in water and mainly consists of a mosaic of two dominating vegetation types; Atlantic heathlands and *Nardus* grasslands[1]. Approximately 200 individuals of free-ranging garranos, one of the oldest pony breeds originating from Portugal, live in this field site (Figure S1b)[2–4]. The horses are mostly living without any human care, however some of them are owned by local residents and they sometimes capture them, especially the young males. The horses are under predation pressure from Iberian wolves (*Canis lupus signatus*) in this region [5]. During mating and breeding seasons, roughly from April to July, multiple harems and AMUs stay in the observation site which we call Zone 1 and Zone 2. From 2014 to 2018, we have identified 324 horses in this field site (including zero-year-old infants). The field sites are a flat area extending about 1 km^2 each and covered with a complex mosaic of heathlands and grasslands, with some rock outcrops and linear elements such as narrow streams of water and artificial tracks. We separated Zones 1 and 2 because they were divided by rocky hills and we rarely observed horses moving between the boundary during the daytime (see results).

Details of the drone operations

We launched drones from more than about 30 m away from horses and retrieved it to where it had launched. We located ourselves to where we can see as many horses as possible, and changed home location as horses migrated. No specialized launching/retrieval equipment was required. Before the first observation, we flew a drone over the whole field site and recorded which horses were available in the field each day (Figure S1a). We recorded horses outside of the field site when these horses were within about 200 m from other horses inside the field site. We conducted operation with two people. Usually one took sequential photographs above horses, while the other recorded the approximate position of horses in a note to support the identification in orthomosaic images. When a herd became spread, we used two drones and each of us

recorded roughly half of the areas to reduce the duration of each observations. During the 30 minutes interval of the observations, we observed the field with binoculars and recorded when horses appeared to/disappeared from the field site.

Sequential photos were taken by a camera deployed to a drone in regular RGB images (equipped with 12.35 MP sensor, 4000 × 3000 pixels). Focus, shutter speed, and IOS were set automatically. We took the sequential photos every 1-2 second/s so that forward overlap of each images became 60% at least. The data were stored on board in a microSD card. We watched real-time images from a drone by using an application 'DJI GO4' (https://www.dji.com/jp/downloads/djiapp/dji-go-4) in iPad mini.

The relationship of the inter-unit distance and direct interaction

We decided the threshold of inter-unit interaction as $p_2 = 122.1$ m. To investigate this threshold was meaningful to the inter-unit relationships, we examined the direct interaction among units.

Most of the interaction we observed were a 'ritual' among males, which was the behavioural sequence starting from running up with each other, followed by sniffing, tossing of the head while squealing and striking with their force legs[6], and sometime it escalated to the actual fighting including kicking, biting and chasing. We were able to identify a stallion ritual from the aerial photos because of the unusual proximity between males from different units.

While males were engaging themselves in the ritual, the females in the males' harem usually did not care and kept foraging. Thus, we considered the distance between the females represent the inter-unit distance when the males recognized each other and started the ritual.

We observed 17 male-male interaction, 6 of them occurred between harem males and the rest occurred between a harem male and a bachelor male. The mean ± SD of the inter-harem distance was 55.9 ± 31.9 m (range 22.4 – 102.4 m). Although the sample size was small, 122.1 m roughly matched with the maximum distance of the interaction, thus we considered it is appropriate to use for the threshold.

Comparing the spatial structure of two periods.

We compared the network of two periods: June 6th – July 4th (Period 1, all units were observed in Zone 2) and July 5th – July 10th (Period 2, units were observed in both Zone 1 and 2). The average distance matrix of these two periods show quite high correlation matrix, where $r = 0.99$ ($p < 0.001$). Then, we averaged interindividual distances in each day, and calculated the correlation index for all pairs of the daily average distance matrix. According to the Mantel test with 9,999 replicates, all of them showed 5% level significance (Figure S2a). Each correlation index of each dyad was separated into three categories: Period1-1 (when both of the days were in Period1), Period1-2 (when one was in Period1 and the other is in Period2), and Period2-2 (when both were in Period2). Kruskal-Wallis rank sum test showed significant difference among these three at $p < 0.01$ level ($\chi^2 = 50.51$; Figure. S2b). Post hoc Dunn's test showed that the correlation of Period1-1 is higher than that of Period1-2 and Period2-2 ($p < 0.01$). We further compared the nearest unit distance in two period using Welch's test. The distance to nearest units were significantly smaller during Period 1 (mean ± SD: 35.0 ± 32.3 m) than Period 2 (54.4 ± 100.8m; $t(980.53) = 5.77$, $p < 0.001$)

Were the units with larger centrality located closer to the herd center?

According to the network analysis, AMUs had lower centrality than harems and larger units had higher centrality than smaller ones. We examined that larger harems were actually located in the central positions, while smaller harems and AMUs were in the periphery of the herd.

We selected the days that all the units were available (June 14th, 18th, 20th, 21st, 23rd), and in total 69 observation were used. We considered the average position of all the units as the centre of the herd. We calculated the distance from it and scored the rank (units with smaller rank is closer to the centre) for each unit in each observation. We first calculated the correlations of the distance from the center and the rank toward strength centrality and both showed negative correlations ($r = -0.66$ for both of the indices). ANOVA and post-hoc Tukey HSD test were also conducted under R environment to examine the distance from the center and the rank differ according to the unit size and type (harem or bachelor). ANOVA showed significant difference in both distance and rank among the categories (distance from the centre: $F(8) = 12.34$, $p < 0.001$; rank $F(8) = 14.7$, $p < 0.001$). Tukey's test indicated that AMUs and smaller harems tended to have larger distance from the centre and higher ranks (Figure S3).

Reference

1. Gonçalves, J. *et al.* Evaluating an unmanned aerial vehicle-based approach for assessing habitat extent and condition in fine-scale early successional mountain mosaics. *Appl. Veg. Sci.* **19**, 132–146 (2016).
2. Ringhofer, M. *et al.* Comparison of the social systems of primates and feral horses: data from a newly established horse research site on Serra D'Arga, northern Portugal. *Primates* **58**, 479–484 (2017).
3. Inoue, S., Yamamoto, S., Ringhofer, M., Mendonça, R. S. & Hirata, S. Lateral position preference in grazing feral horses. *Ethology* **00**, 1–9 (2019).
4. Ringhofer, M. *et al.* Herding mechanisms to maintain the cohesion of a harem group: two interaction phases during herding. *J. Ethol.* 1–7 (2019). doi:10.1007/s10164-019-00622-5
5. Álvares, F., Domingues, J., Sierra, P. & Primavera, P. Cultural dimension of wolves in the iberian peninsula: Implications of ethnozoology in conservation biology. *Innovation* **24**, 313–331 (2011).
6. Feh, C. Relationships and communication in socially natural horse herds. in *The domestic horse: The origins, development and management of its behaviour* (eds. Mills, D. S. & McDonnell, S. M.) 83–93 (Cambridge University Press, 2005).
7. R Core Team. *R: A language and environment for statistical computing.* (R Foundation for Statistical Computing, 2019).
8. Hothorn, T., Bretz, F., Westfall, P. & Heiberger, R. M. Package 'multcomp' Title Simultaneous Inference in General Parametric Models. *Biometrical J.* **50**, 346–363 (2016).

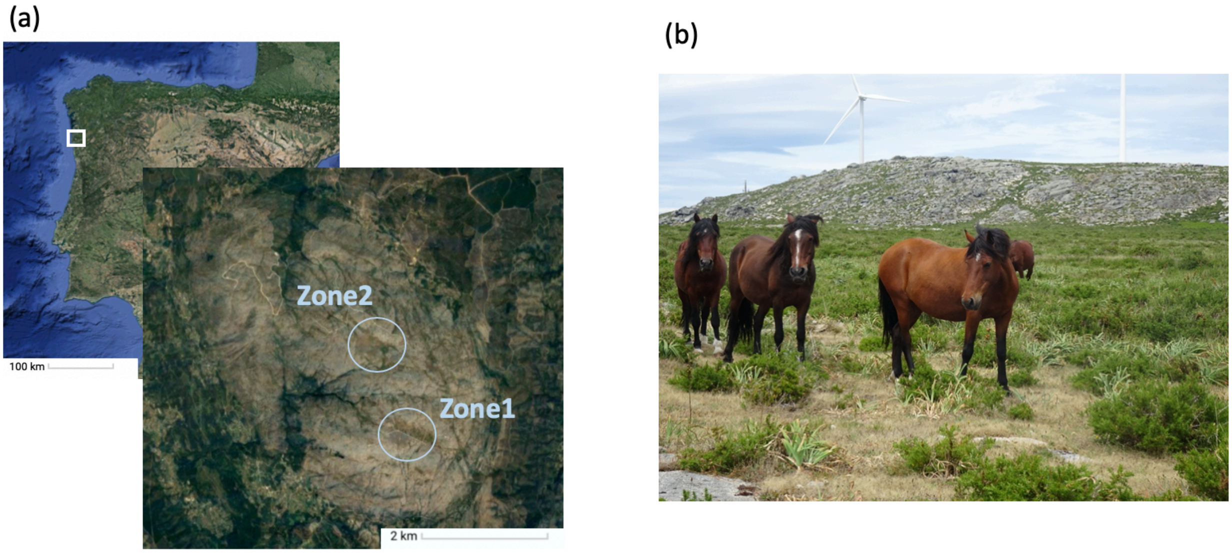

Figure S1. (a) The location of the study site, Serra D'Arga. The photos were obtained from Google map (https://www.google.co.jp/maps/). (b) The photograph of feral horses, breeds named garranos, in Serra D'Arga. The photo was taken by the authour (T. Maeda).

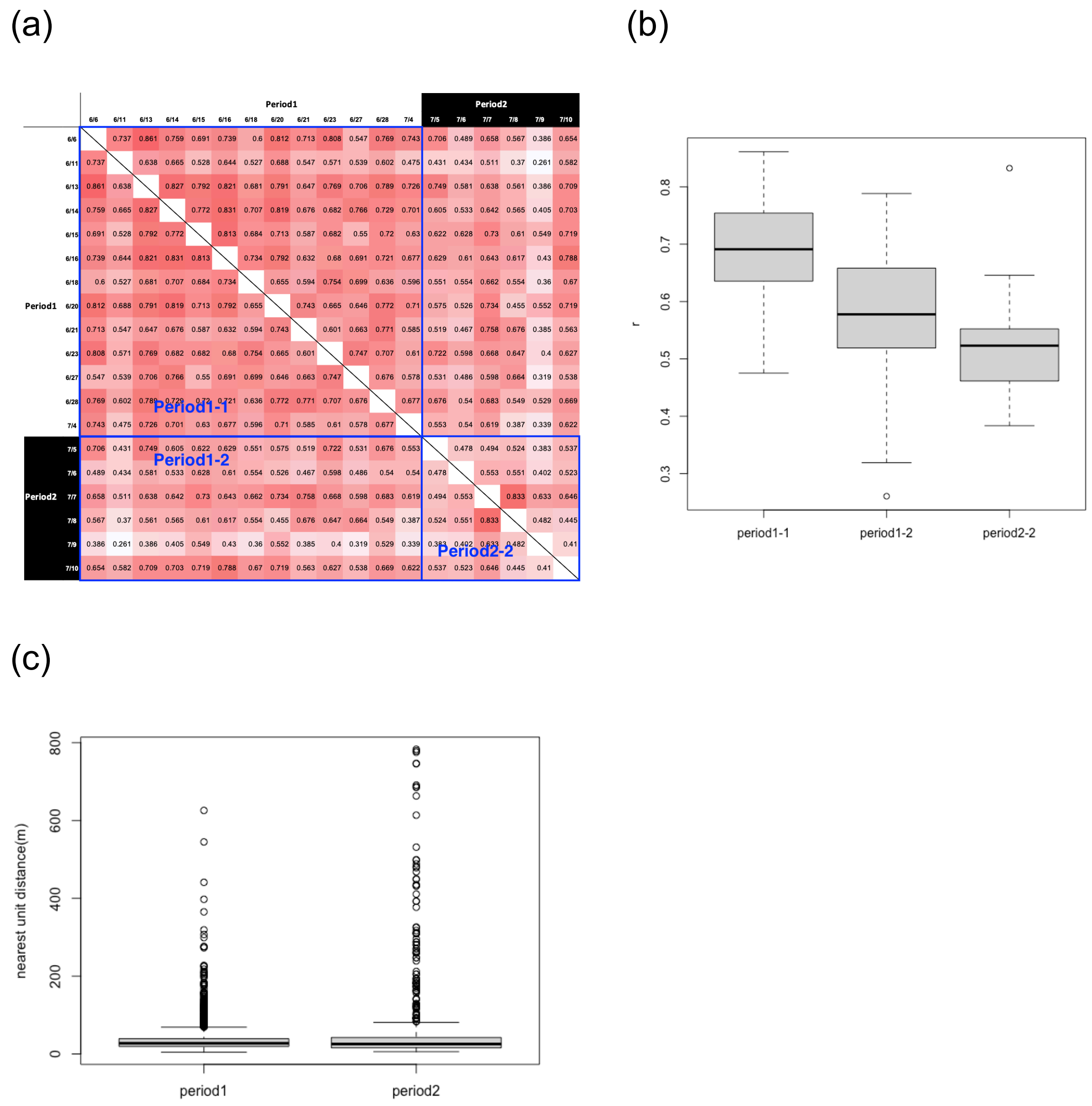

		Period1													Period2					
		6/6	6/11	6/13	6/14	6/15	6/16	6/18	6/20	6/21	6/23	6/27	6/28	7/4	7/5	7/6	7/7	7/8	7/9	7/10
Period1	6/6		0.737	0.861	0.759	0.691	0.739	0.6	0.812	0.713	0.808	0.547	0.769	0.743	0.706	0.489	0.658	0.567	0.386	0.654
	6/11	0.737		0.638	0.665	0.528	0.644	0.527	0.688	0.547	0.571	0.539	0.602	0.475	0.431	0.434	0.511	0.37	0.261	0.582
	6/13	0.861	0.638		0.827	0.792	0.821	0.681	0.791	0.647	0.769	0.706	0.789	0.726	0.749	0.581	0.638	0.561	0.386	0.709
	6/14	0.759	0.665	0.827		0.772	0.831	0.707	0.819	0.676	0.682	0.766	0.729	0.701	0.605	0.533	0.642	0.565	0.405	0.703
	6/15	0.691	0.528	0.792	0.772		0.813	0.684	0.713	0.587	0.682	0.55	0.72	0.63	0.622	0.628	0.73	0.61	0.549	0.719
	6/16	0.739	0.644	0.821	0.831	0.813		0.734	0.792	0.632	0.68	0.691	0.721	0.677	0.629	0.61	0.643	0.617	0.43	0.788
	6/18	0.6	0.527	0.681	0.707	0.684	0.734		0.655	0.594	0.754	0.699	0.636	0.596	0.551	0.554	0.662	0.554	0.36	0.67
	6/20	0.812	0.688	0.791	0.819	0.713	0.792	0.655		0.743	0.665	0.646	0.772	0.71	0.575	0.526	0.734	0.455	0.552	0.719
	6/21	0.713	0.547	0.647	0.676	0.587	0.632	0.594	0.743		0.601	0.663	0.771	0.585	0.519	0.467	0.758	0.676	0.385	0.563
	6/23	0.808	0.571	0.769	0.682	0.682	0.68	0.754	0.665	0.601		0.747	0.707	0.61	0.722	0.598	0.668	0.647	0.4	0.627
	6/27	0.547	0.539	0.706	0.766	0.55	0.691	0.699	0.646	0.663	0.747		0.676	0.578	0.531	0.486	0.598	0.664	0.319	0.538
	6/28	0.769	0.602	0.789	0.729	0.72	0.721	0.636	0.772	0.771	0.707	0.676		0.677	0.676	0.54	0.683	0.549	0.529	0.669
	7/4	0.743	0.475	0.726	0.701	0.63	0.677	0.596	0.71	0.585	0.61	0.578	0.677		0.553	0.54	0.619	0.387	0.339	0.622
Period2	7/5	0.706	0.431	0.749	0.605	0.622	0.629	0.551	0.575	0.519	0.722	0.531	0.676	0.553		0.478	0.494	0.524	0.383	0.537
	7/6	0.489	0.434	0.581	0.533	0.628	0.61	0.554	0.526	0.467	0.598	0.486	0.54	0.54	0.478		0.553	0.551	0.402	0.523
	7/7	0.658	0.511	0.638	0.642	0.73	0.643	0.662	0.734	0.758	0.668	0.598	0.683	0.619	0.494	0.553		0.833	0.633	0.646
	7/8	0.567	0.37	0.561	0.565	0.61	0.617	0.554	0.455	0.676	0.647	0.664	0.549	0.387	0.524	0.551	0.833		0.482	0.445
	7/9	0.386	0.261	0.386	0.405	0.549	0.43	0.36	0.552	0.385	0.4	0.319	0.529	0.339	0.383	0.402	0.633	0.482		0.41
	7/10	0.654	0.582	0.709	0.703	0.719	0.788	0.67	0.719	0.563	0.627	0.538	0.669	0.622	0.537	0.523	0.646	0.445	0.41	

Figure S2. (a) The correlation of the average distance matrix of each day and (b) its boxplot. The color of the cell represents the level of correlation (red is higher and white is lower). (c) Nearest inter-unit distance of two periods. The table was created under Microsoft Excel for Mac 16.16.25 (https://www.microsoft.com/en-us/microsoft-365/excel) and the box plots were created under R 3.6 environment[7].

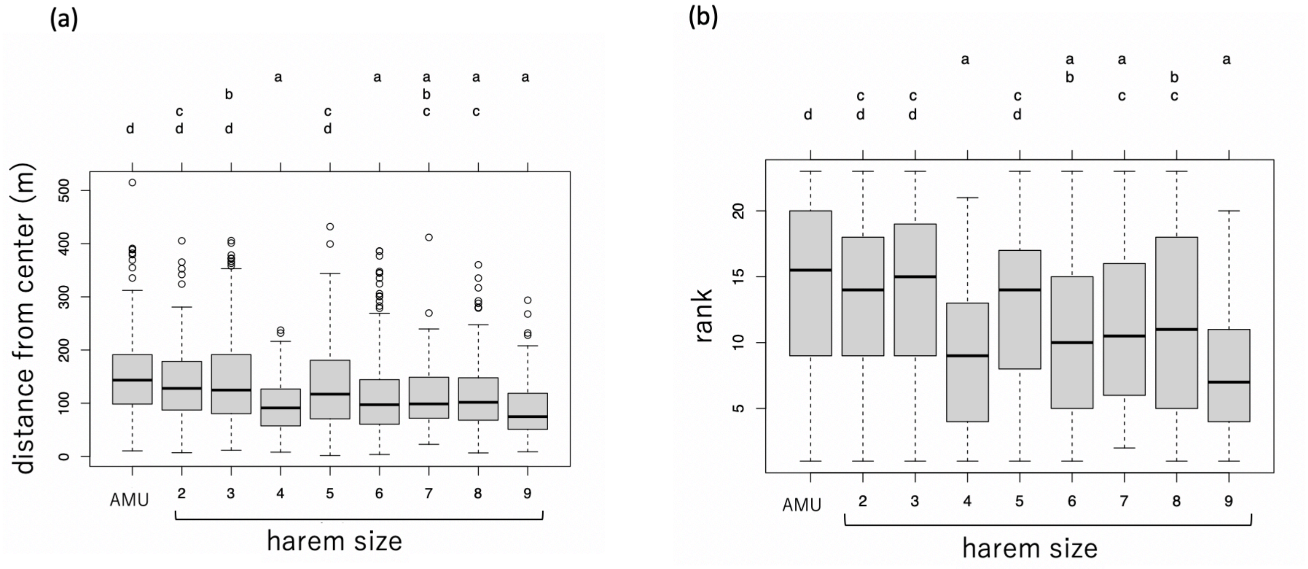

Figure S3. The results of ANOVA and post-hoc Tukey tests of (a) distance from the center and (b) the rank. Higher ranks mean the unit tends to be in the peripheral area of the herd. When two categories have different alphabets above the graph (for example, “a” and “c”), they have significant difference each other at 5% significance level. The graphs were created using R package ‘multcomp’[8].

Date	June												July							FO
	6	11	13	14	15	16	18	20	21	23	27	28	4	5	6	7	8	9	10	
**Number of harem**	**8**	**13**	**19**	**21**	**21**	**21**	**21**	**21**	**21**	**21**	**19**	**20**	**14**	**19**	**17**	**17**	**9**	**12**	**18**	**0.83**
Asaka	3	3	3	3	3	3	3	3	3	3	3	3	3	3	3	3	3	3	3	1.00
Aso		4	4	4	4	4	4	4	4	4	4	4	4	4	4	4			4	0.84
Bizen	2	2	2	2	2	2	2	2	2	2	2	2		2	2	2	2	2	2	0.95
Gozen_Nagaoka	5	5	5	5	5	5	5	5	5	5	5	5	5	5	5	5	5	5	5	1.00
Hakata		5	5	5	5	5	5	5	5	5	5	5		5	5	5			5	0.79
Harajuku			8	8	8	8	8	7*	8	8	8	8	8	8	8	8			8	0.74
Hitachi	6	6	6	6	6	6	6	6	6	6	6	6	6	6	6	6			6	0.89
Hokuei			6	6	6	6	6	6	6	6	6	6	6	6	6				6	0.74
Kamakura_Zama			6	6	6	6	6	6	6	6	6	6		6	6	6			6	0.74
Kanoya		3	3	3	3	3	3	3	3	3	3	3	3	3	3	3	3	3	3	0.95
Kanuma				7	7	6**	6	6	6	6	***									0.32
Kitakami				8	8	8	8	8	8	8	8	8	8	8	8	8	8	8	8	0.84
Kobe	9	9	9	9	9	9	9	9	9	9	9	9	9	9	9		9	9	9	0.95
Nagoya			6	6	6	6	6	6	6	6	6	6	6	6	6	6	6	6	6	0.89
Nanba_Tennoji		7	7	7	7	7	7	7	7	7	7	7		7	7	7		7	7	0.84
Numazu		6	6	6	6	6	6	6	6	6		6	6	6			6	6	6	0.79
Seki	2		2	2	2	2	2	2	2	2	2	2				2		2		0.68
Taiji		6	6	6	6	6	6	6	6	6	6	6	6	6	6	6	6	6	6	0.95
Taka matsu		6	6	6	6	6	6	6	6	6	6	6	6	6	6	6		6	6	0.89
Takaoka_Uozu	5	5	5	5	5	5	5	5	5	5	5	5	5	5	5	5			5	0.89
Tsu	4		4	4	4	4	4	4	4	4	4	4		4		4				0.68
**Number of AMU**	**1**	**2**	**1**	**2**	**1**	**1**	**2**	**2**	**2**	**2**	**1**	**1**	**1**	**2**	**1**	**1**	**1**	**1**	**2**	**0.71**
AMU1		5		5			5	5	5	5		5		5	5		5	5	5	0.63
AMU2	2	2	2	2	2	2	2	2	2	2	2		2	2		2			2	0.79
Hirosaki	1	1	1	1	1	1	1	1	1	1	1	1	1	1	1	1			1	0.89
Gujyo					1		1	1	1	1	1		1	1		1			1	0.53
Hyuga								1	1	1										0.16
Unnan						1				1		1	1			1				0.26
Yamaga			1	1								1	1	1						0.26

Figure S4. The availability of the unit groups during observations in a daily basis. The number of the cells represent the number of non-adult horses in a unit. When the cell is yellow, it represents that a focal unit appeared in the field. FO means the frequency of occurrence. Hirosaki, Gujyo, Hyuga, Unnan, and Yamaga are solitary males. *A female Machida did not appear. ** A female, Oyama, disappeared (maybe predated, or caught by humans). *** Kanuma group has not been observed since then until now (July, 2020). The table was created under Microsoft Excel for Mac 16.16.25 (https://www.microsoft.com/en-us/microsoft-365/excel).

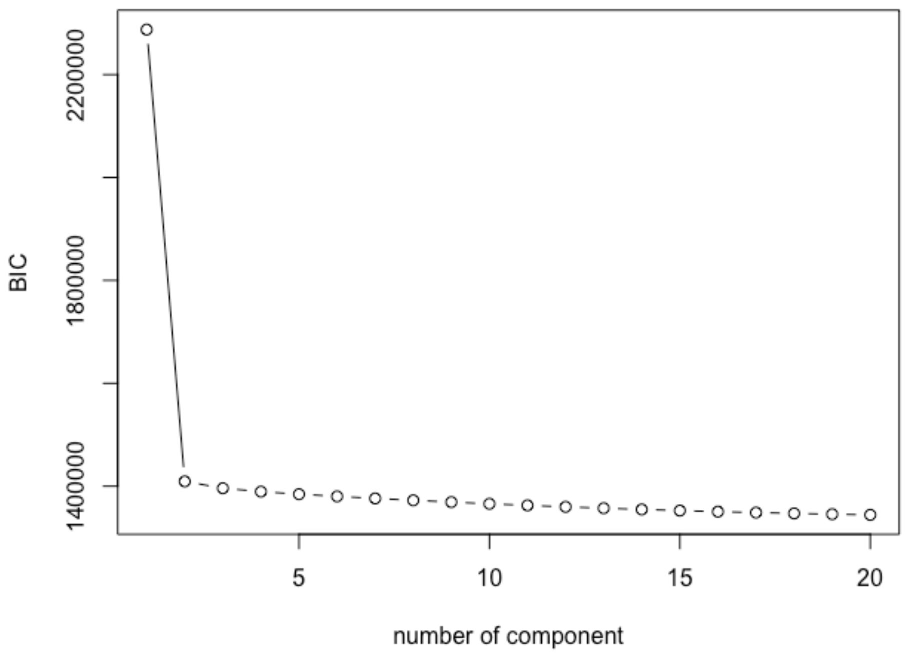

Figure S5. The Bayesian information criterion (BIC) of the Gaussian mixture model with different number of components (k).

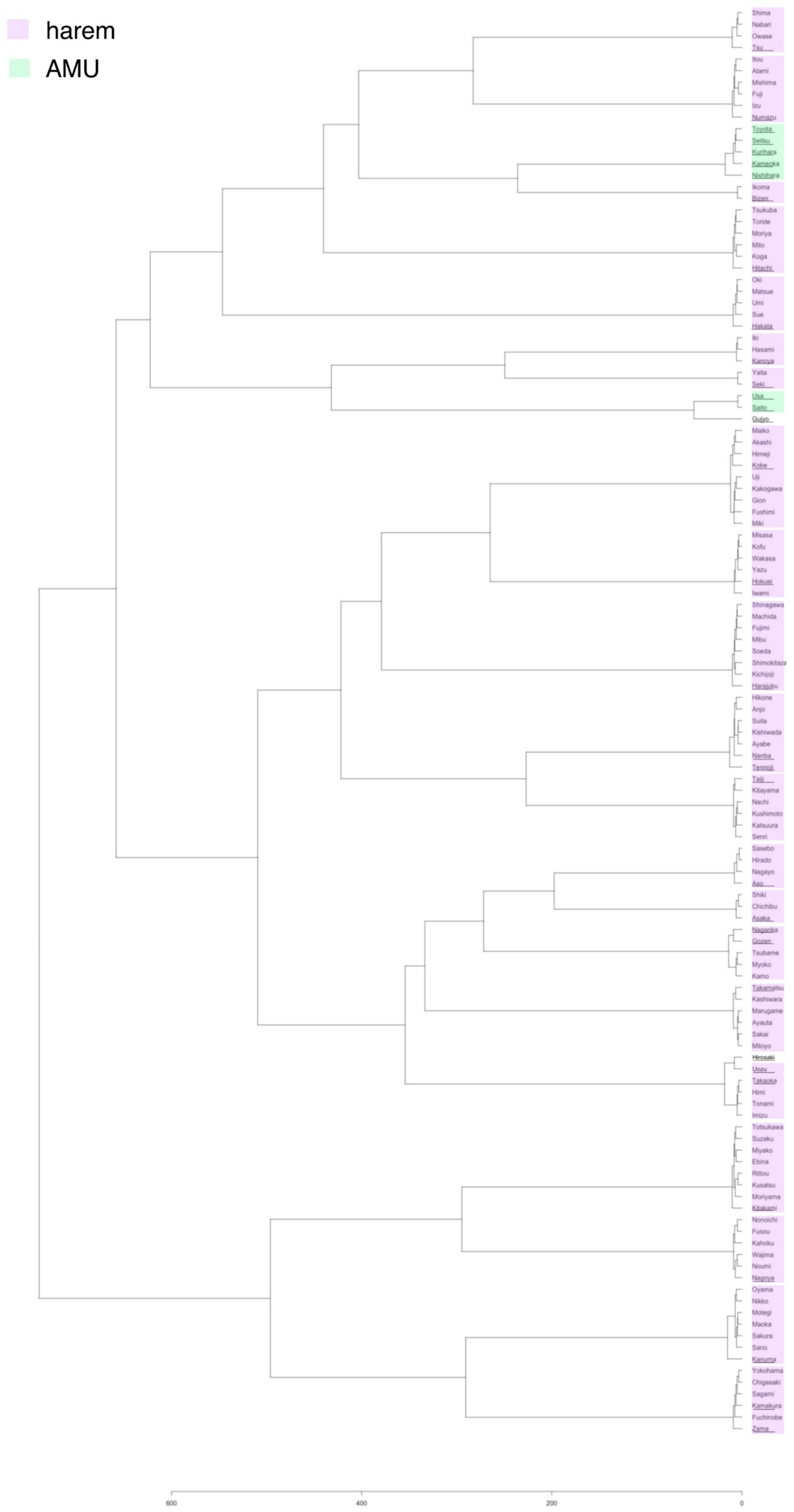

Figure S6. Dendrogram of the horses, drawn based on the average inter-individual distances using Ward method under R environment. We excluded three solitary males because they did not appear in the field very often. The pink and green box represent the unit partition we have made, where the pink is harems and the green is AMUs. The underlined individuals are adult males. The graph was created under R 3.6 environment[7].

Table S1. Property of units. We named the harems from the ID of harem males, and we joint two names like "male1-male2" in case of two-male harems. Group size includes only adults and young.

group type	group name	size	adult		young		infant	
			male	female	male	female	male	female
harem	Bizen	**2**	1	1	0	0	0	0
	Seki	**2**	1	1	0	0	0	0
	Asaka	**3**	1	2	0	0	0	0
	Kanoya	**3**	1	2	0	0	0	0
	Aso	**4**	1	3	0	0	0	1
	Tsu	**4**	1	3	0	0	1	1
	Hakata	**5**	1	3	0	1	0	0
	Gozen-Nagaoka	**5**	2	3	0	0	0	1
	Takaoka-Uozu	**5**	2	2	0	1	0	1
	Hitachi	**6**	1	3	1	1	0	2
	Hokuei	**6**	1	5	0	0	0	0
	Kanuma	**6***	1	5*	0	0	0	1
	Nagoya	**6**	1	5	0	0	0	0
	Numazu	**6**	1	4	0	1	0	0
	Taiji	**6**	1	5	0	0	0	0
	Takamatsu	**6**	1	4	0	1	1	0
	Kamakura-Zama	**6**	2	4	0	0	1	0
	Nanba-Tennoji	**7**	2	5	0	0	1	0
	Harajuku	**8**	1	7	0	0	1	3
	Kitakami	**8**	1	7	0	0	1	1
	Kobe	**9**	1	7	0	1	2	0
**Total**		**113***	**25**	**81**	**1**	**6**	**8**	**11**
AMU	AMU1	**5**	5	-	-	-	-	-
	AMU2	**2**	2	-	-	-	-	-
**Total**		**7**	**7**					

*Kanuma group consisted of 7 individuals until they lost a female sometime between June 15th and 16th.
